# Graphic representation of the burden of suffering in dizziness patients

**DOI:** 10.1186/s12955-014-0184-2

**Published:** 2014-12-19

**Authors:** Steffi Weidt, Annette Beatrix Bruehl, Hanspeter Moergeli, Dominik Straumann, Stefan Hegemann, Stefan Büchi, Michael Rufer

**Affiliations:** Department of Psychiatry and Psychotherapy, University Hospital Zurich, Culmannstrasse 8, 8091 Zurich, Switzerland; Department of Psychiatry, Psychotherapy and Psychosomatics, University Hospital of Psychiatry Zurich, Zürich, Switzerland; Behavioural and Clinical Neuroscience Institute and Department of Psychiatry, University of Cambridge, Cambridge, UK; Department of Neurology, University Hospital Zurich, Zürich, Switzerland; Department of Otolaryngology, Head & Neck Surgery, University Hospital, Zurich, Switzerland; Department for Psychosomatics, Clinic for Psychotherapy and Psychosomatics Hohenegg, Meilen, Switzerland

**Keywords:** Burden of suffering, Dizziness, Emotional distress, Graphic representation, PRISM

## Abstract

**Background:**

Dizziness adversely affects an individual’s well-being. However, its impact is not only influenced by its physical manifestations, but also by its subjective importance to the patient. Appropriately assessing the subjective burden of dizziness is difficult. The Pictorial-Representation of Illness- and Self-Measure (PRISM), on which patients illustrate the distance between their ‘self’ and their illness, has been documented to indicate the perception of suffering in several different illnesses. Our study objectives were (1) to assess how useful the PRISM is in patients with dizziness; and (2) to determine which clinical, emotional and sociodemographic factors contribute to their burden of suffering.

**Methods:**

A total of 177 outpatients with dizziness completed this cross-sectional study, in which the following measures were assessed of suffering rated using the PRISM tool; dizziness-related variables, like emotional distress (Hospital Anxiety and Depression-Scale, HADS); self-perceived severity of dizziness (Dizziness Handicap Inventory, DHI); and sociodemographic variables.

**Results:**

Regression analyses identified the strongest association between PRISM-rated suffering and DHI (p < 0.001), explaining 34% of the variance in PRISM-rated suffering. The HADS score and having continuous dizziness versus transient attacks each explained roughly 2% of the variance in suffering. No significant associations with PRISM-rated suffering were found for sociodemographic variables or other dizziness characteristics.

**Conclusions:**

The PRISM is applicable to patients suffering from dizziness, demonstrating a significant association with the severity of dizziness and reliably distinguishing between those with low and high intensities of dizziness. The PRISM also reflects the multi-factorial aspects of suffering. Due to its immediate, timesaving and economical use, the PRISM could enable clinicians to identify vulnerable patients at risk for chronic symptoms and distress. Whether the PRISM can detect improvements and worsening of symptoms during treatment warrants further research.

## Background

Dizziness is one of the most frequent complaints in medical care; one that can negatively and significantly affect a patient’s well-being [1-3]. Measuring the impact of an illness is important, because it helps to identify those patients at risk for chronic symptoms and distress. One multi-faceted concept pertinent to measuring the impact of dizziness is the assessment of health-related quality of life (HRQoL). HRQoL takes into account different components of the patient’s current life situation and is widely used to determine the impact of illness on an individual’s well-being [4]. In patients with dizziness, HRQoL seems to be independent of other measurable characteristics of the dizziness, like the duration of symptoms and objective balance tests [4-8]. The self-perceived HRQoL of patients with dizziness is significantly impaired relative to that of the general population, regardless of the aetiology of dizziness [4-6,9-12]. While dizziness is more common in women than men [2,13], gender does not appear to influence the HRQoL of patients with dizziness [4,14]. However, employment status and education are known to be associated with HRQoL [15,16]. Factors like being in a stable relationship and living with someone are also generally associated with better HRQoL [4,17,18].

While there seem to be good insights into the impact of dizziness on patient self-perceived HRQoL [19], the burden of suffering from dizziness has not yet been investigated. One important facet of the overall burden of illness is the burden of suffering due to the illness [20]. Burden of suffering is defined as ‘a state of severe distress associated with events that threaten the intactness of the person’ [21]. It can be quickly and easily rated by patients themselves using a simple visual instrument called the *Pictorial Representation of Illness and Self- Measure* (PRISM). [22], which has been documented to reliably assess the burden of suffering in various disease states like orofacial pain, chronic urticaria, systemic lupus erythematosus, psoriasis, post-traumatic stress disorder, rheumatoid arthritis, and chronic obstructive pulmonary disease [23-28].

Since the patient’s self-perceived severity of illness and their perception of the burden of suffering, measured with the PRISM, have been found to be strongly correlated [27,29,30], burden of suffering seems to be an important indicator of an individual’s well-being and the PRISM a reliable, feasible and useful outcome measure in the management of illness [31].

Despite the importance of the burden of suffering, previous studies have rarely focused upon burden in patients with dizziness. Moreover, to our knowledge, how applicable the PRISM is in patients with dizziness remains non-investigated. Therefore, the current study aimed to investigate whether the self-administered PRISM can be used to assess the burden of suffering in patients with dizziness. In addition, we aimed to investigate which factors in these patients (e.g., sociodemographic) contribute to the burden of suffering, as measured with the PRISM. The underlying hypotheses were (1) that the PRISM score and perceived severity of dizziness are significantly correlated; and (2) that this relationship is robust, even when adjusted for other factors on multi-variate analysis. We also expected the PRISM to encompass different aspects of the dizziness experience, like perceived dizziness severity and level of emotional distress.

## Methods

### Sample

This research received no grants from any funding agency in the public, commercial or non-profit sectors. The study is part of a cross-sectional research project investigating patients with dizziness [4]. It was approved by the ethics committee of the Canton of Zurich, Switzerland. All participants gave their written informed consent prior to data collection. From August 2010 to August 2011, we asked patients between the ages of 18 and 65 years, inclusive, who had been referred to the Interdisciplinary Centre for Vertigo and Balance Disorders at University Hospital Zurich to participate in the study. They received questionnaires by mail and were asked to return them at the time of their clinical consultation. Patients were excluded from the study if the PRISM tool was missing.

### Measures

**Figure 1:**
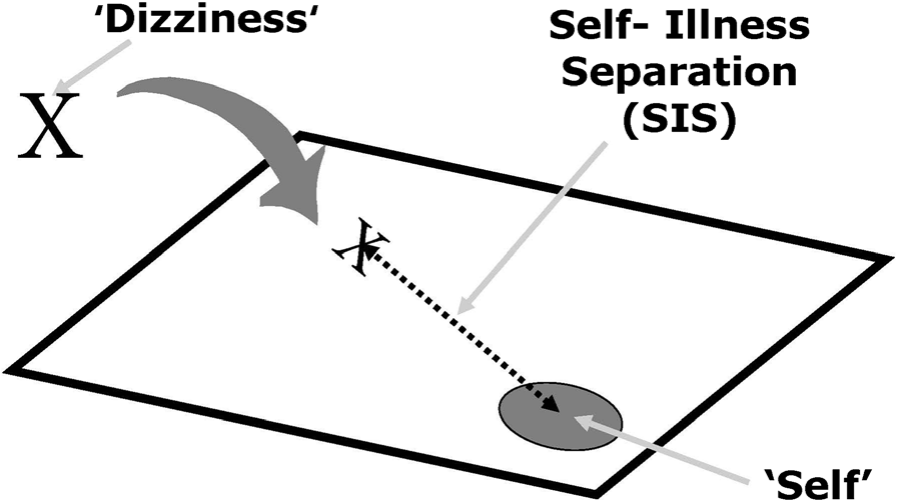
**PRISM, paper-pencil version.** Shows the paper-and-pencil version of the PRISM used in the current investigation. The circle represents the Self. The cross marks the position of dizziness in relation to the Self; the line represents the SIS (Self illness separation) measured in millimetres. Patients were given the following written instructions: “The box on the paper represents your current life and the circle in the lower right hand corner represents you. Where would you mark the dizziness in your life at the moment?” The closer the cross is positioned relative to the self-circle centre, the higher the indicated burden of suffering from dizziness.

The *Pictorial Representation of Illness and Self Measure* (PRISM) is a very brief and simple visualization tool that patients can use to rate their burden of suffering [20]. With the PRISM, patients depict the distance between their illness and themselves by positioning a disc within a rectangular field in which a circle in one corner represents the person’s self [22]. The distance between the illness and the self (the distance between the centres of the two discs) is called *self-illness-separation* (SIS), which is a direct measure of a patient’s perception of the controllability of their illness, and an inverse measure of its intrusiveness; in other words, the smaller the SIS, the greater the self-perceived intrusiveness of disease [26]. In this way, it is also inversely correlated with self-perceived of suffering, in that a small SIS indicates high levels of suffering. The PRISM has been successfully validated and used as an outcome measure for a variety of clinically-distinct diseases and health states, including lung diseases [26,32,33], rheumatoid arthritis [20,26,34,35], systemic lupus erythematosus [24-26], multiple sclerosis [22], grief and trauma [22,27,28,33], pain [23,36], shared decision making [37], risk perception [38], cancer [33,39,40], dermatological diseases [29,30,33,41], fertility problems [33], and diabetes [42]. For our study, the paper and pencil version was used. The measurement derived from the PRISM is the self-illness- separation (SIS), which ranges from 0 to 90 mm (the distance between the patient’s pencil mark and the centre of the self-circle). In terms of interpretation, two SIS ranges were distinguished (a mark within the self-circle, and a mark outside the self-circle) [26]. Previous studies have identified no disadvantages of the paper and pencil versus the original version, which uses a disc instead of a pencil mark [23,43,44]. The test and its instructions are shown in Figure 1.

The *Dizziness Handicap Inventory* (DHI, German version) is a disease-specific, self-rating questionnaire that assesses the patient’s severity of dizziness [45,46]. It consists of 25 items, each having 3 response options (yes = 4, sometimes = 2, no = 0) and total scores ranging from 0 to 100. Three sub-scales measure the *functional* (DHIF, 9 items; e.g., Does your problem interfere with your job or household responsibilities?), *physical* (DHIP, 7 items; e.g., Does walking down the aisle of a supermarket increase your problems?) and *emotional* (DHIE, 9 items; e.g., Because of your problem, do you feel frustrated?) impact of dizziness. The German version has demonstrated good internal consistency (α = 0.72 to 0.89) and reliability (test-retest reliability r = 0.92 to 0.97) and thus is recommended as a measure of symptom severity in patients with dizziness [11,46].

The *Hospital Anxiety and Depression Scale* (HADS, German version) was used to assess symptoms of emotional distress [47,48]. Each of the 14 items is rated on a scale from 0 to 3, resulting in a summation score between 0 and 42 [49]. The scale has been shown to be an effective measure of emotional distress with acceptable test-retest reliability among patients with a vestibular disorder [50,51].

Clinical and socio-demographic characteristics were assessed with a questionnaire that had been developed for clinical use at the Interdisciplinary Centre for Vertigo and Balance Disorders at University Hospital Zurich.

### Statistical analysis

Descriptive statistics were calculated as means with standard deviations, or as percentages. The relationships between SIS, DHI, the three DHI subscales, HADS score, and clinical characteristics were analysed using Pearson correlation coefficients. Further correlation analyses were conducted to evaluate the relationships between SIS and additional variables (e.g., age). To compensate for multiple testing, a Bonferroni correction was applied to the results of the 24 correlations, the threshold for significance thereby adjusted to 0.05/24 = 0.002. Inter-group comparisons of SIS for different nominal variables like gender were performed using Student’s t-tests and one-way analysis of variance (ANOVA). Hierarchical regression analyses were conducted to measure the influence of different variables on SIS variance, with SIS as the dependent variable. All variables identified as statistically significant on bivariate analysis were entered into the models. The DHI total score model was constructed by introducing (variable insertion method: Enter) the following variables step by step into the model: a) DHI; b) DHI and continuous dizziness vs. transient attacks (code: 0 continuous, 1 transient attacks) and c) DHI, continuous dizziness vs. transient attacks and HADS score. The DHI subscale model was similarly constructed by introducing the following variables step by step into the model: a) DHI subscales; b) DHI subscales and continuous dizziness vs. transient attacks; and c) DHI subscales, continuous dizziness vs. transient attacks, and the HADS score. The Durbin-Watson test for hierarchical regression (1.76 and 1.84) suggested independent errors [35]. The average variance inflation factor was not substantially greater than 1.0 (between 1.1 and 1.6), suggesting that the regression was not biased by multicollinearity [36,37]. To compare patients who marked their dizziness inside their self-circle against those who marked it outside, t-tests were performed. The threshold for statistical significance was set as p < 0.05, unless otherwise specified (e.g., adjusting for multiple testing). All calculations were performed using the statistical software-package SPSS (version 22).

## Results

### Subject characteristics (N = 177)

Two hundred and three patients seen at the Interdisciplinary Centre for Vertigo and Balance Disorders at University Hospital Zurich provided their written consent and agreed to complete both the questionnaires and the PRISM tool. However, of these 203 patients, 26 neglected to complete the PRISM, leaving 177 subjects for analysis. These 177 did not differ from the 26 excluded for a missing PRISM in terms of mean DHI total score, HADS score, age, duration of symptoms, or gender distribution (all p > 0.1, data not shown). The mean age of participants was 44.4 years, 86 (48.6%) were male, and the mean SIS was 27.5 mm. The mean duration of dizziness was 161.9 weeks. Testing for skewness (3.5) indicated a non-symmetrical distribution with data skewed to the right (median: 55.5 weeks, IQR: 24–173). Further socio-demographic and clinical characteristics are shown in Table 1.

**Table 1:** **Demographic and clinical characteristics of 177 patients with dizziness**

	**Mean**	**SD**
Age, years	44.4	11.9
PRISM, SIS in mm	27.5	20.5
DHI total score	46.5	23.4
DHIF	17.2	10.2
DHIP	13.9	7.3
DHIE	15.2	8.8
HADS	13.7	8.1
Duration of dizziness, weeks	161.9	271.9
	**Number of patients**	**%**
Gender, female/male	91/86	51.4/48.6
Partnership, yes/no	134/41	75.7/23.2
Employment status, yes/no	124/49	70.1/27.7
**Education**		
No degree or basic school education	27	15.2
Apprenticeship or high school diploma	106	59.9
University degree	41	23.2
**Characteristics of dizziness**		
Continuous/Transient attacks	54/102	30.5/57.6
Vertigo	42	23.7
Non-vertigo	13	7.3
Mixed (vertigo and non-vertigo)	120	67.8

PRISM: Pictorial Representation of Illness and Self Measure; SIS: Self-Illness-Separation; DHI: Dizziness Handicap Inventory; DHIF: DHI functional scale; DHIP: DHI physical scale; DHIE: DHI emotional scale; HADS: Hospital Anxiety and Depression Scale; Partnership: Living with someone (married and not married).

### Association between the PRISM (SIS) and clinical and socio-demographic characteristics

SIS was inversely correlated with the DHI total score, DHI sub-scores, and the HADS score (all p < 0.001). These correlations remained significant even after Bonferroni correction. SIS did not correlate with the duration of dizziness symptoms (Spearman’s rho = −0.05; p = 0.54) or age (r = 0.03; p = 0.70) (Table 2). Vertigo characteristics of dizziness and SIS exhibited significant associations, but did not survive Bonferroni correction.

**Table 2:** **Bivariate correlations between SIS and clinical characteristics**

	**PRISM; SIS**	**HADS**	**DHI total**
PRISM; SIS	1		
HADS	-.44***	1	
DHI total	-.56***	.56***	1
DHIF	−0.51***	0.50***	0.95***
DHIP	−0.41***	0.34***	0.80***
DHIE	−0.57***	0.60***	0.88***
DoD^a^	−0.05	0.003	−0.05
AvP	0.31***	−0.04	−0.25**
VNV^b^	F = 3.25*	4.20*	4.34*
age	0.03	−0.04	−0.02

Pearson correlations unless otherwise specified; ^a^Spearman’s correlation coefficient; ^b^ANOVA; ***p < 0.001; **p < 0.01;*p < 0.05; Bonferroni-corrected threshold for significance: 0.002; PRISM: Pictorial Representation of Illness and Self- Measure; SIS: Self illness separation; DHI total: Dizziness Handicap Inventory, total summation score, DHIF: DHI functional scale; DHIP: DHI physical scale; DHIE: DHI emotional scale; HADS: Hospital Anxiety and Depression scale; DoD: duration of dizziness; AVP: transient attacks (coded as 1) versus continuous dizziness (coded as 0); VNV: vertigo versus non-vertigo versus mixed (vertigo and non-vertigo) dizziness.

There were no differences in mean SIS between female and male subjects, or between those living with someone and those living alone. There also were no associations between SIS and either employment status or level of education (Table 3).

**Table 3:** **Association between SIS and socioeconomic characteristics**

**Condition**	**Mean in mm (SD)**	**t** ^**1**^ **; (F)** ^**2**^	**df** ^**1**^ **, (df1,df2)** ^**2**^	**p**
Gender^1^		−1.17	175	0.24
Female	25.8 (18.7)			
Male	29.4 (22.2)			
Partnership^1^		−0.68	173	0.50
Yes	27.1 (20.4)			
No	29.6 (20.6)			
Employment^1^		1.39	171	0.17
Yes	28.7 (21.5)			
No	23.9 (17.8)			
Education^2^		(0.84)	(2, 171)	0.33
No degree or basic school education	26.9 (17.9)			
Apprenticeship or high school diploma	26.8 (20.4)			
University degree	31.6 (21.9)			

SIS: Self-Illness-Separation, SD: standard deviation, Partnership: Living with someone (married and not married), ^1^Student’s t-test; ^2^one-way ANOVA.

Forty-five of the 177 patients placed their dizziness mark inside their self-circle (low SIS, high suffering). These patients reported significantly more severe dizziness than patients who placed the mark outside their self-circle (DHI total: t = 5.19; p < 0.001, mean difference = 19.62; SE difference = 3.78). DHIF, DHIP, and DHIE also were significantly higher in patients who marked their dizziness inside their self-circle versus those who marked it outside (mean difference from 4.1 to 8.6, all p ≤ 0.001). In addition, these 45 patients suffered from significantly greater emotional distress (HADS: t = 4.24; p < 0.001, mean difference = 7.57; SE difference = 1.36).

### PRISM variance (SIS)

When total DHI score was introduced alone into the hierarchical regression model, it explained roughly thirty-four percent of the variance in SIS. When, in the second and third models, whether dizziness was continuous or transiently episodic and the HADS score were included, each variable explained an additional two percent of SIS variance. In the third model, severity of dizziness and SIS variance exhibited the strongest association (β = −0.45; p < 0.001). Continuous dizziness vs. transient attacks and the HADS rating exhibited smaller but still significant associations as well (Table 4).

**Table 4:** **Hierarchical regression model summaries for PRISM (SIS) with DHI total score**

**Model summaries (method: ENTER)**					**Model parameters for model 3**		
**Models**	**Adjusted R** ^**2**^	**R** ^**2**^ **change**	**F change**	**Sig F change**	**Variables**	**Beta**	**p(Beta)**
**1**	0.34	0.34	77.4	<0.001	DHI	−0.45	<0.001
**2**	0.36	0.02	5.6	0.02	AvP	0.18	0.01
**3**	0.37	0.02	4.4	0.04	HADS	−0.17	0.04

Model 1: DHI; Model 2: model 1 + AvP; Model 3: model 2 + HADS.

PRISM: Pictorial Representation of Illness and Self Measure; SIS: Self-Illness-Separation; AvP: leading dizziness characteristic (0 continuous, 1 transient attacks); HADS: Hospital Anxiety and Depression Scale summation score, DHI: Dizziness Handicap Inventory total score.

On DHI sub-score analysis, during which DHI sub-scores DHIF, DHIP, and DHIE were entered into the regression model, roughly thirty-nine percent of SIS variance was explained by these variables, with DHIE as the only significant variable (p ≤ 0.01). The second and third models, again introducing continuous versus transient dizziness and the HADS rating, also contributed significantly to SIS variance (Table 5), with small contributions from both variables.

**Table 5:** **Hierarchical regression model summaries for PRISM (SIS) with DHI subscales**

**Model summaries (method: ENTER)**					**Model parameters for model 3**		
**Models**	**Adjusted R** ^**2**^	**R** ^**2**^ **change**	**F change**	**Sig F change**	**Variables**	**Beta**	**p(Beta)**
**1**	0.39	0.41	32.0	<0.001	DHIF	−0.15	0.21
**2**	0.42	0.03	6.1	0.02	DHIP	−0.03	0.73
**3**	0.43	0.02	5.0	0.03	DHIE	−0.34	≤0.01
**-**	-	-	-	-	AvP	0.19	≤0.01
**-**	-	-	-	-	HADS	−0.18	0.03

Model 1: DHI subscales; Model 2: model 1 + AvP; Model 3: model 2 + HADS.

PRISM: Pictorial Representation of Illness and Self Measure; SIS: Self illness separation; AvP: leading dizziness characteristic (0 continuous, 1 transient attacks); HADS: Hospital Anxiety and Depression Scale summation score, DHI: Dizziness Handicap Inventory subscales, DHIF: DHI functional scale; DHIP: DHI physical scale; DHIE: DHI emotional scale.

## Discussion

The current study aimed to investigate how applicable the PRISM instrument is in patients with dizziness. The burden of suffering measured with the PRISM and patients’ self-perceived severity of dizziness were expected to be significantly associated. We also sought to differentiate between additional factors influencing the burden of suffering in these patients. As expected, a high burden of suffering (low SIS) was significantly associated with a high self-perceived severity of dizziness (high DHI). Furthermore, patients with a high burden of suffering (low SIS) reported more emotional distress (high HADS) and were more likely to report continuous dizziness than transient attacks.

Before discussing the implications of our results and their relationship to previously-reported findings, we feel it necessary to discuss our study’s limitations. First, burden of suffering does not necessarily imply anything about specific, helpful coping strategies that an individual might use to deal with his or her disease. Suffering among patients with dizziness still needs to be investigated in the context of coping to identify those strategies most helpful at reducing the burden of suffering. Other individual influences, like alexithymia, should be investigated as well [14]. Furthermore, our study provided only limited information about the underlying cause of dizziness, because it included no objective tests or diagnostic procedures. It is possible that different underlying causes affect SIS scores and their associations with other variables.

It has previously been shown that affective distress potentially influences the burden of an illness and contributes to the multi-factorial construct of the PRISM [22]. In our sample, a high burden of suffering, as reflected by a low SIS, was directly correlated with self-perceived severity of dizziness (DHI). On regression analysis approximately thirty-four percent of SIS variance was explained by the severity of dizziness. Furthermore, our results demonstrated a significant correlation between emotional distress and SIS. For both, the DHI total score and DHI sub-score regression analysis, the HADS rating accounted for an additional two percent of SIS variance.

The association between a subjective measure of illness (DHI) and SIS is consistent with other studies which found the PRISM to reflect the subjective features of illness (e.g., pain, depression, grief) [1,28]. However, only a small number of previous studies [24,25,28,40] have examined whether the PRISM construct and the subjective construct of the illness are identical, or related but not identical. While certain other studies have supported our finding of a multi-factorial construct of the PRISM, reflected by the association between SIS and HADS score on bivariate analysis [26-28], another study identified no such association on multivariate analysis [24]. However, this latter study did not include HADS as an overall measure of emotional distress, but used sub scores for depressive and anxiety symptoms. As the HADS measures overall emotional distress [50] and does not reliably differentiate between depressive and anxiety symptoms, we decided to use the scale as an overall measure of distress. Nevertheless, the additional variance explained by the HADS in the current study seems rather low. One potential explanation relates to the structure of the DHI and HADS, which both encompass aspects of emotional distress. This overlapping measurement of emotional distress might underestimate the role of the HADS. This view is supported by our DHI sub-score analyses, in which the emotional subscale of the DHI emerged as the strongest predictor. However, it seems that self-perceived severity of dizziness might fail to encompass the full nature of the burden of suffering in patients with dizziness. This assumption is further supported by the significant association between SIS and continuous dizziness on multivariate analysis.

The current results also suggest that the burden of suffering is not gender-specific in patients with dizziness, which is consistent with previous results that identified no significant gender differences in SIS [42]. The burden of suffering also was not correlated with age in the current sample. However, one might expect to measure a higher burden of suffering in older patients, due to co-morbid conditions that occur more often in the elderly. The current results and results of one previous study [24] do not confirm this expectation. To the contrary, perhaps due to greater life experiences, older patients might have learned how to cope better with various life stressors [25,42]. Overall, socioeconomic variables played no crucial role in the burden of suffering in patients with dizziness, which strengthens previous impressions that the PRISM might be less influenced by socioeconomic and cultural factors than other subjective assessments [29].

Nevertheless, our data illustrated, that PRISM-rated suffering is likely to yield a more comprehensive picture of the individual’s illness experience than the Dizziness Handicap Inventory (DHI). Thus, the PRISM seems to assess a multi-factorial construct of suffering in patients with dizziness. This finding is consistent with both our *a priori* expectations and previously-published studies on PRISM [26]. Taken together, our study suggests that suffering, as measured with the PRISM, is a significant multi-factorial component of well-being that contributes to and, thus, aids in the understanding of the burden of dizziness.

In our study, the PRISM was used as a self-report instrument with no observer guidance. Nevertheless, meaningful results that supported our hypotheses were obtained. In addition, the PRISM reliably distinguished between patients with mild versus severe dizziness. As such, the PRISM could enable clinicians to assess the severity of dizziness symptoms easily, and help them to identify patients at risk for chronic distress relating to their dizziness, who might warrant further consultations and psychological support. Therefore, we suggest a timesaving and economical stepwise assessment of patients with dizziness. This stepwise approach could include the PRISM as an initial screening instrument. Should this screening suggest a high burden of suffering, further examination of symptom severity and distress should be under-taken. Furthermore, the PRISM seems to reflect a patient’s appraisal of the intrusiveness and controllability of their symptoms [26]. This might point to the PRISM not only as a screening tool, but also as a tool to evaluate the effectiveness of treatment [27].

## Conclusions

The current study clarified some of the relevant contributors to the burden of suffering, and therefore provides a more complete picture of illness burden in patients with dizziness. Our results also show that the PRISM tool captures certain aspects of the illness experience, like emotional distress and self-perceived severity of dizziness. As such, we see the PRISM as a multi-factorial measure of burden of suffering in these patients that is relatively independent of sociodemographic and cultural influences. In addition, the PRISM seems to reliably distinguish between patients with less and more severe dizziness. Therefore, it could enable clinicians to identify vulnerable patients at risk for developing chronic symptoms and distress, thereby interfering with daily living. Whether the PRISM can be used to evaluate improvement or worsening of symptoms during treatment will require future studies.

## Abbreviations

PRISM: Pictorial-representation of illness- and self-measure
SIS: Self- illness- separation
HRQoL: Health-related quality of life
DHI: Dizziness handicap inventory
DHIF: DHI functional subscale
DHIP: DHI physical subscale
DHIE: DHI emotional subscale
HADS: Hospital anxiety and depression scale
DoD: Duration of dizziness
